# Sustainable management of peanut damping-off and root rot diseases caused by *Rhizoctonia solani* using environmentally friendly bio-formulations prepared from batch fermentation broth of chitinase-producing *Streptomyces cellulosae*

**DOI:** 10.1186/s12870-024-05441-6

**Published:** 2024-08-09

**Authors:** Gaber Attia Abo-Zaid, Mai H. Darwish, Hanan A. Ghozlan, Muhammad A Abdel-Gayed, Soraya A. Sabry

**Affiliations:** 1 Bioprocess Development Department, Genetic Engineering and Biotechnology Research Institute (GEBRI), City of Scientific Research and Technological Applications (SRTA-City), New Borg El-Arab City, Alexandria 21934 Egypt; 2 Botany and Microbiology Department, Faculty of Science, Alexandria University, Alexandria, 21526 Egypt; 3 Onion, Garlic and Oil Crops Diseases Research Department, Plant Pathology Research Institute, Agricultural Research Center, Giza, 12619 Egypt

**Keywords:** *Rhizoctonia solani*, *Streptomyces cellulosae*, Chitinase, Fermentation, Bio-formulation, Biocontrol

## Abstract

**Background:**

Soil-borne plant diseases represent a severe problem that negatively impacts the production of food crops. Actinobacteria play a vital role in biocontrolling soil-borne fungi.

**Aim and objectives:**

The target of the present study is to test the antagonistic activity of chitinase-producing *Streptomyces cellulosae* Actino 48 (accession number, MT573878) against *Rhizoctonia solani*. Subsequently, maximization of Actino 48 production using different fermentation processes in a stirred tank bioreactor. Finally, preparation of bio-friendly formulations prepared from the culture broth of Actino 48 using talc powder (TP) and bentonite in a natural as well as nano forms as carriers. Meanwhile, investigating their activities in reducing the damping-off and root rot diseases of peanut plants, infected by *R*. *solani* under greenhouse conditions.

**Results:**

Actino 48 was found to be the most significant antagonistic isolate strain at *p* ≤ 0.05 and showed the highest inhibition percentage of fungal mycelium growth, which reached 97%. The results of scanning electron microscope (SEM) images analysis showed a large reduction in *R*. *solani* mycelia mass. Additionally, many aberrations changes and fungal hypha damages were found. Batch fermentation No. 2, which was performed using agitation speed of 200 rpm, achieved high chitinase activity of 0.1163 U mL^− 1^ min^− 1^ with a yield coefficient of 0.004 U mL^− 1^ min^− 1^ chitinase activity/g chitin. Nano-talc formulation of Actino 48 had more a significant effect compared to the other formulations in reducing percentages of damping-off and root rot diseases that equal to 19.05% and 4.76% with reduction percentages of 60% and 80%, respectively. The healthy survival percentage of peanut plants recorded 76.19%. Furthermore, the nano-talc formulation of Actino 48 was sufficient in increasing the dry weight of the peanut plants shoot, root systems, and the total number of peanut pods with increasing percentages of 47.62%, 55.62%, and 38.07%, respectively.

**Conclusion:**

The bio-friendly formulations of actinobacteria resulting from this investigation may play an active role in managing soil-borne diseases.

## Background

Peanut, groundnut (*Arachis hypogaea* L.), is one of the greatest imperative oilseed crops in the world. Damping-off and root rot are two plant diseases, which are caused by *Rhizoctonia solani.* They are considered the main soil-borne fungal diseases of peanut. Managing this fungal pathogen is very difficult because it lives on plant debris and in the soil for extensive period of time in a form of sclerotia. Also, it has a wide range of host plants such as sugar beet, tomato, and cucumber [1]. Chemical pesticides (agrochemicals), which are used in controlling soil-borne fungal diseases are the cause of several environmental pollution problems, have encouraged the agro-products producers and farmers to use the biological control strategies as alternative approaches for sustainable agriculture [2]. The usage of biocontrol agents can play a vital role in reducing plant diseases and maintaining the natural balance within an existing ecological system [3]. Actinobacteria are considered strong biocontrol agents that show high antagonistic effects against diverse plant diseases [4]. Two main functions were suggested for actinobacteria that enable them to work as biocontrol agents. These functions are colonization of plant roots surface and production of various secondary metabolites that interfere fungal growth [5, 6]. For example, actinobacteria have various properties as saprophytic soil bacteria such as production of antibiotics, secondary metabolites, and chitinolytic enzymes [7]. Actinobacteria represent 90% of chitinolytic microorganisms. Various *Streptomyces* spp. have been characterized as an important producers of chitinolytic enzymes such as *S*. *plicatus*, *S*. *lividans*, *S*. *virdificans*, *S*. *halstedii* and *S*. *champvatii* [8]. Through the last two decades, actinobacteria, particularly *Streptomyces* spp., were recognized as hopeful promising microbial biocontrol agents for their aptitude to produce chitinase enzymes. Sadeghi et al. (2006) [9] used two chitinolytic streptomycetes as promising isolates in inhibiting the mycelial growth of *R*. *solani* of sugar beet. Boukaew et al. (2016) [10] recorded the antifungal activity of the crude and partially purified chitinase enzyme produced by *S*. *philanthi* RM-1-1-38 against *R*. *solani* PTRRC-9 that causes rice sheath blight disease. The in vitro antifungal activity of chitinolytic *S*. *vinaceusdrappus* S5MW2 against *R*. *solani* was detected in a dual culture assay [11]. Lee et al. (2012) [12] demonstrated that the biological control of pepper anthracnose utilizing *S*. *cavourensis* relies on the impact of different chitin-degrading enzymes and an antifungal substance, 2-furancarboxaldehyde. Application of chitinase-producing streptomycetes (two strains) in soil infected by *R*. *solani*, a fungus responsible for sugar beet damping-off disease, inhibited the fungal growth. Additionally, this treatment induced plant growth with an increase in their shoot and root dry weight [9].

Therefore, in the current study, the work aims to achieve the following objectives; primary, estimate the inhibitory effect of some actinobacterial isolates against *R*. *solani*, the main factor in peanut damping-off and root rot diseases. Secondly, use fermentation technology to maximize the production of chitinase-producing *S*. *cellulosae* Actino 48, the hopeful actinobacterial isolate. Ultimately, assess the effectiveness of chitinase-producing *S*. *cellulosae* Actino 48 in reducing damping-off and root rot diseases of peanut plants caused by *R*. *solani* under greenhouse environment using talc and bentonite formulations, both in their natural and nano forms.

## Results

### Antagonistic effect of actinobacterial isolates against *R*. *solani*

**Fig. 1:**
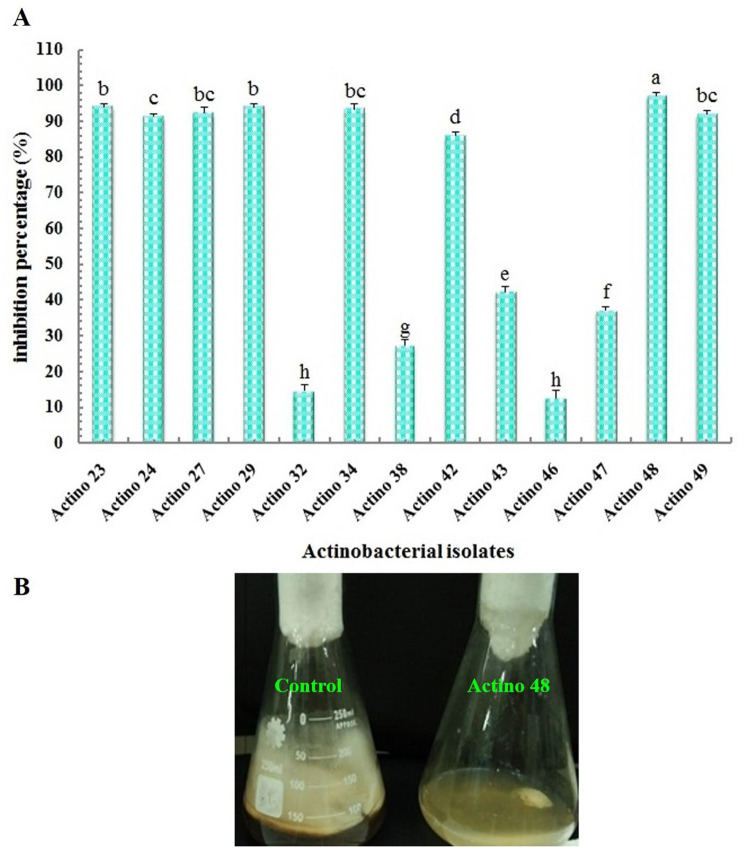
(**A**) Inhibition percentage of actinobacterial isolates against *Rhizoctonia solani* and (**B**) antagonistic effect of actinobacterial isolate Actino 48 against *R. solani*. Flask on the left is the corresponding control (fungus) without the antagonist

Thirteen actinobacterial isolates were tested as potential biological control agents for their antagonistic effect on the in vitro growth of *R*. *solani*. The data obtained in the current study revealed significant differences between actinobacterial isolates. Actino 48 (accession number, MT573878) was the most significant isolate in suppressing the fungal mycelial expansion of *R*. *solani* compared to the other isolates. This isolate recorded the maximum inhibition percentage of 97% against the pathogen followed by actinobacterial isolates Actino 23 and Actino 29, both demonstrated an inhibition percentage of 94% (Fig. 1).

### Assessment of chitinase production by actinobacterial isolates through qualitative and quantitative approaches

**Fig. 2:**
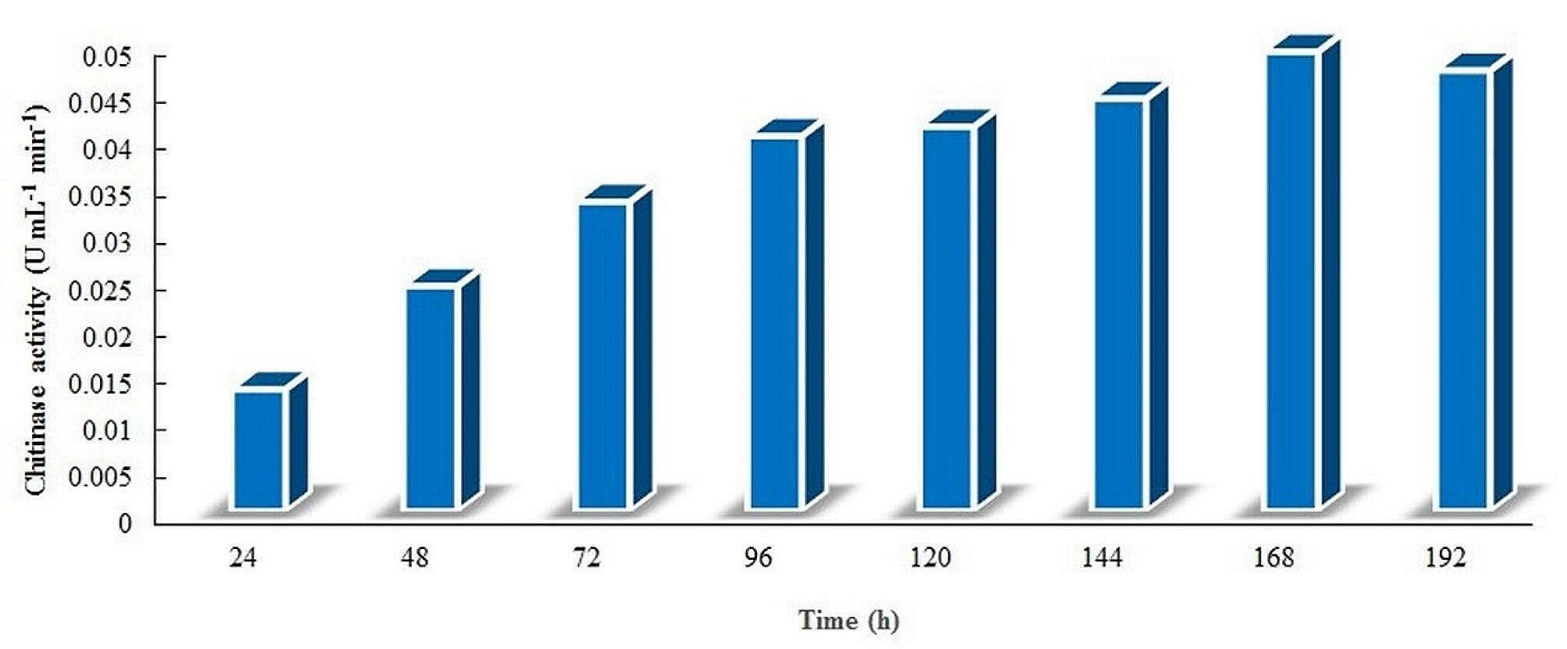
Chitinase activity of cell-free supernatant of *Streptomyces cellulosae* Actino 48

The capability of the actinobacterial isolates to generate chitinase enzyme was assessed in a qualitative manner on a chitin agar plates. Thirteen actinobacterial isolates were utilized in the present investigation. These isolates were able to generate halo zones surrounding the colonies, which demonstrated their ability to produce chitinase and degrade chitin. Among these isolates, Actino 48 was identified as *S*. *cellulosae*, exhibiting the greatest inhibition percentage among all isolates against *R*. *solani* and forming a substantial halo zone around the colony. Thus, this isolate was selected for the production of chitinase enzyme. The maximum chitinase activity was observed at seven days of cultivation (0.049 U mL^*−* 1^ min^*−* 1^). After that, the enzyme activity decreased to reach 0.047 U mL^*−* 1^ min^*−* 1^ at eight days (Fig. 2).

### Detection of interaction between Actino 48 and *R. solani*

**Fig. 3:**
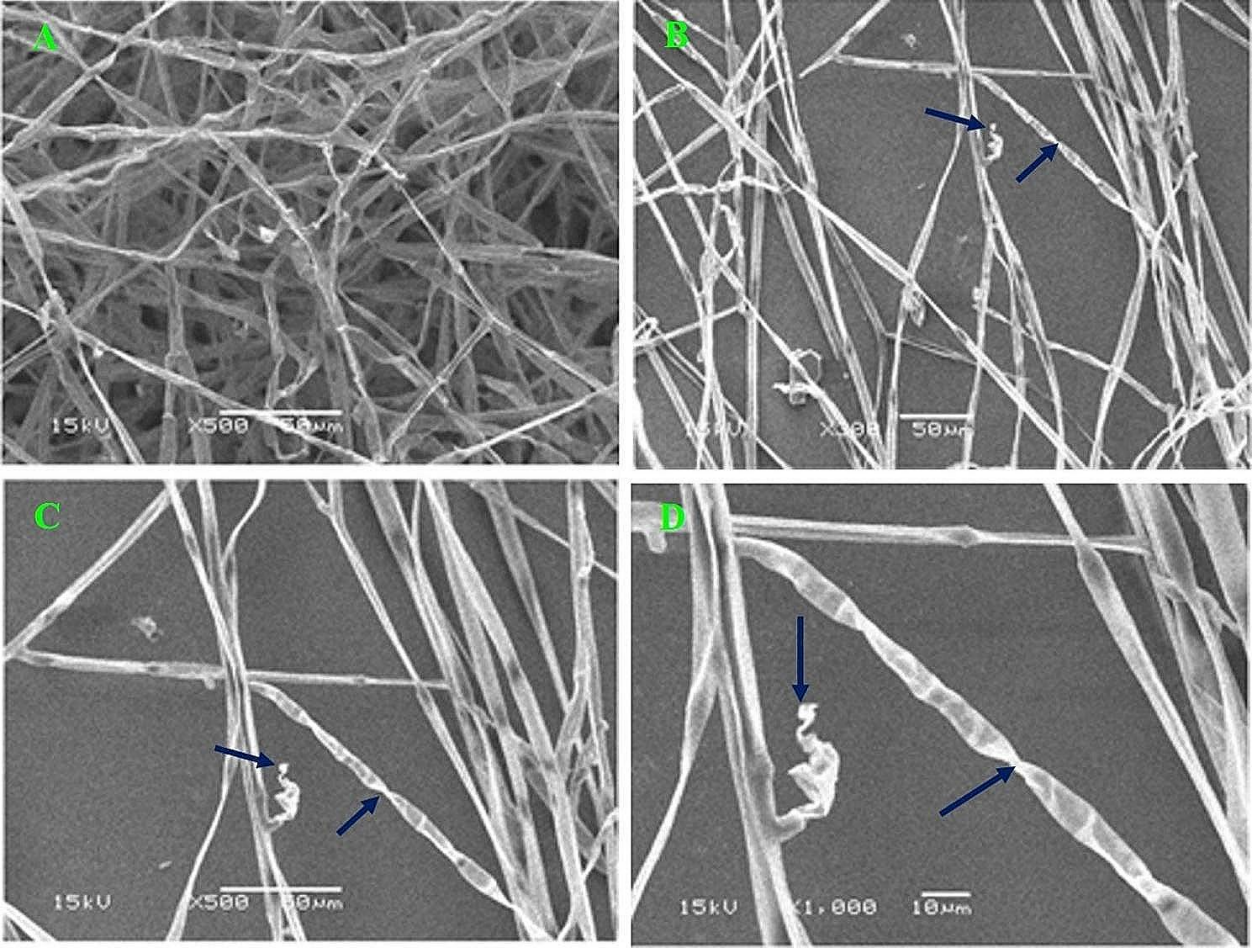
Scanning electron micrographs (SEM) of the antagonistic effect of *Streptomyces cellulosae* Actino 48 against *R. solani*. Micrograph (**A**) is the corresponding fungus control without the antagonistic actinobacterium, micrographs (**B**), (**C**), and (**D**) are the fungus in the presence of the antagonistic actinobacterium with different magnifications. Arrows indicate abnormality and malformation of the fungal hypha of *R. solani*

Scanning Electron Microscope (SEM) micrographs analysis of the interaction between *R*. *solani* and chitinase-producing *S*. *cellulosae* Actino 48, which showed a higher inhibition percentage against *R*. *solani* than other isolates, showed a large reduction in the mass of fungal mycelia and mycelia growth density. Additionally, many aberrations, and damages are shown on the fungal hypha. (Fig. 3).

### Fermentation experiment

#### Batch fermentation

Batch fermentation of *S. cellulosae* Actino 48 was carried out in a 10-L bench-top bioreactor at a constant temperature of 30 °C and uncontrolled pH conditions that was adjusted to pH 6 at the beginning of the fermentation processes.

**Fig. 4:**
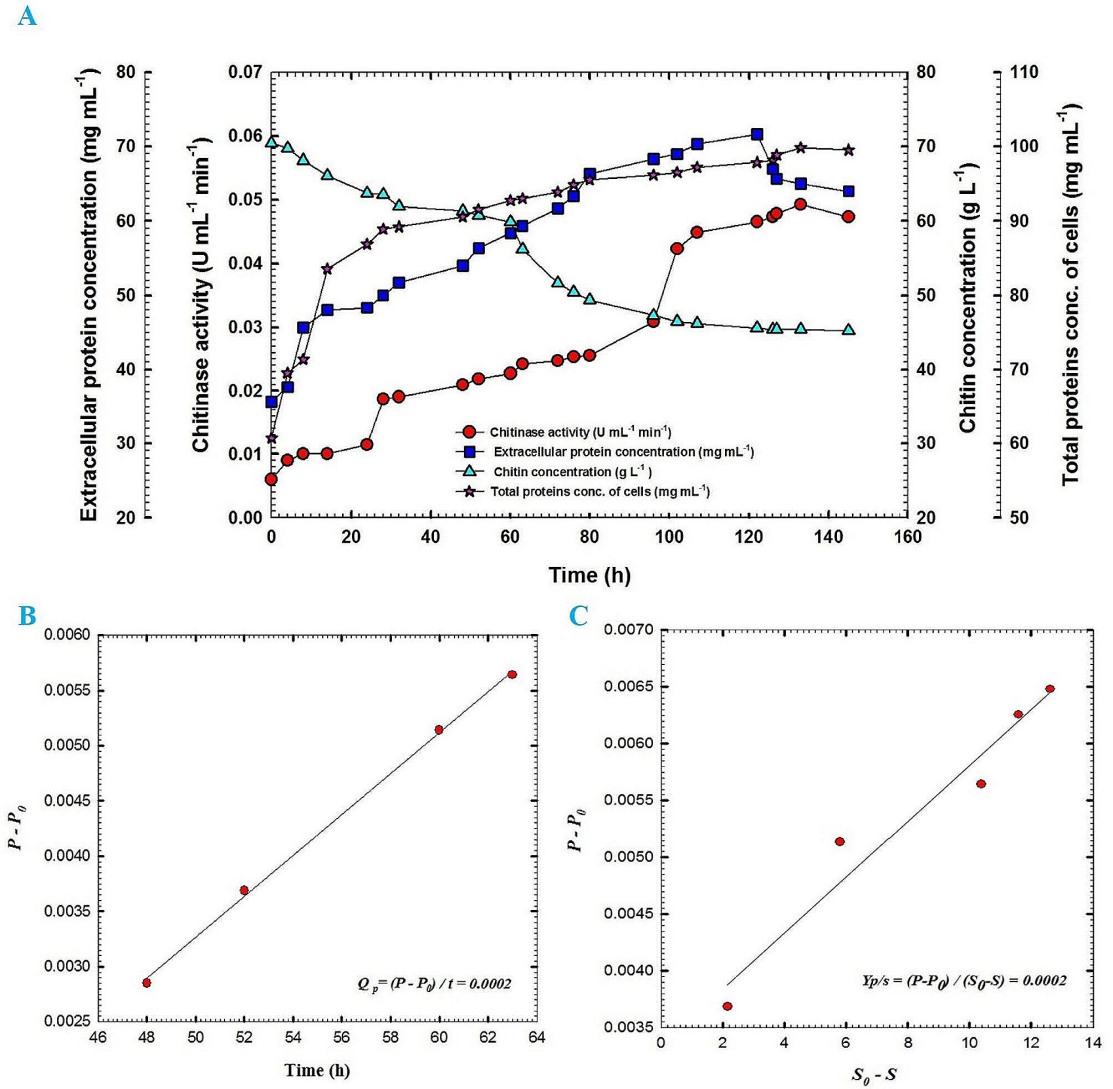
(**A**)Chitinase activity, extracellular protein concentration, chitin concentration and total protein concentration of cells of fermentation broth of *Streptomyces cellulosae* Actino 48 as a function of time for batch fermentation No. 1, (**B**) production rate of chitinase enzyme in the exponential growth phase of batch fermentation No. 1 of Actino 48 and (**C**) yield coefficient of chitinase activity of batch fermentation No. 1 of Actino 48 on chitin; P_0_ represents chitinase activity (U mL^− 1^ min^− 1^) in the broth at initial time t_0_; P, chitinase activity (U mL^− 1^ min^− 1^) in the broth at time t; S_0_, chitin concentration in the broth at initial time t_0_ (g L^− 1^) and S, chitin concentration in the broth at time t (g L^− 1^). Data were analyzed statistically at the significant level of *P* ≤ 0.05

**Fig. 5:**
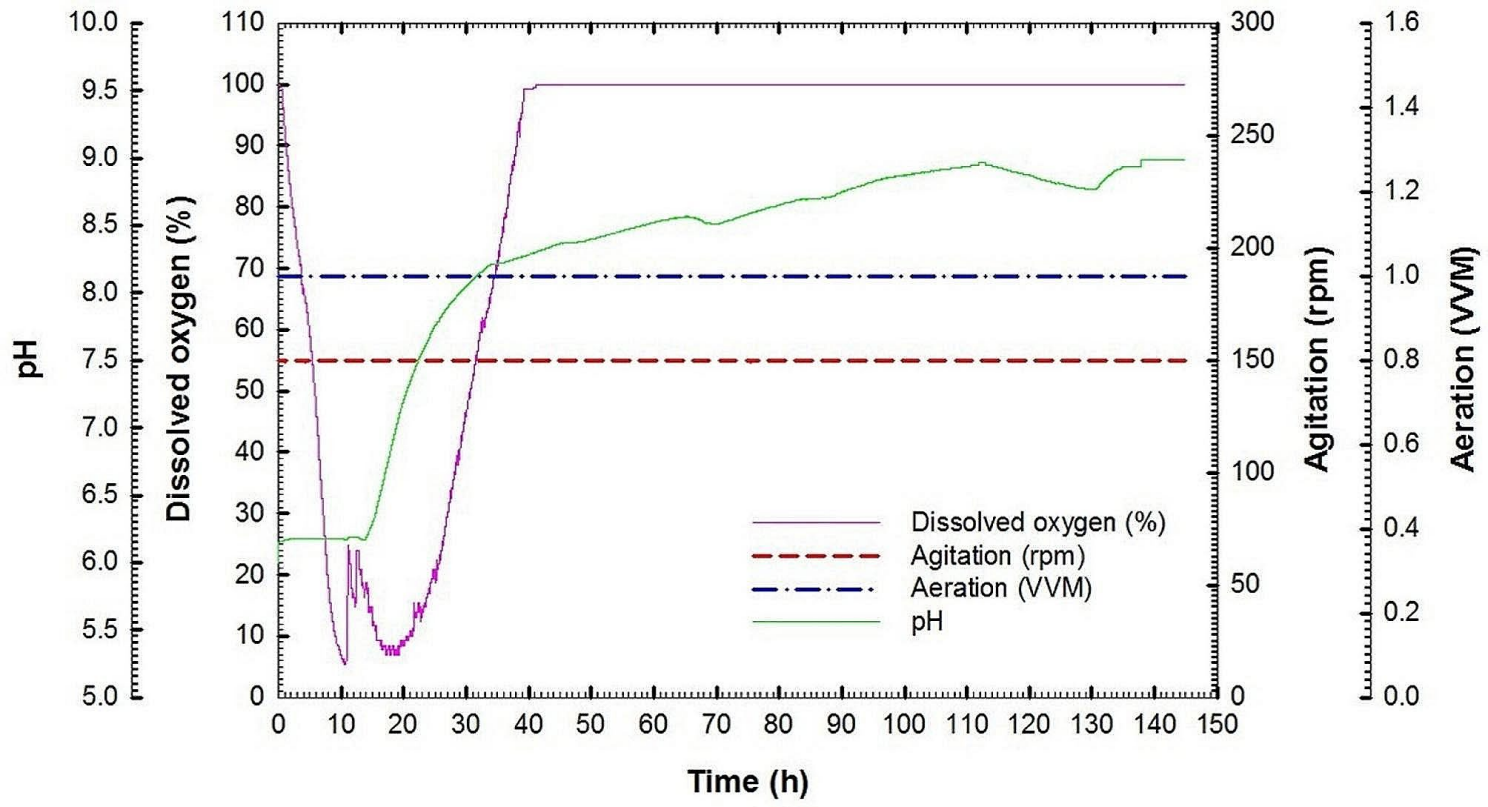
Dissolved oxygen, agitation, aeration and pH (online data) as a function of time during batch fermentation No. 1 of *Streptomyces cellulosae* Actino 48

Batch fermentation No. 1 was carried out at 30 °C with agitation speed and aeration of 150 rpm and 1 VVM, respectively. Figure 4A shows chitinase activity, extracellular protein concentration, chitin concentration, and total protein concentration of actinobacterial cells of culture broth for *S*. *cellulosae* Actino 48 as a function of time. Chitinase activity reached its maximum value of 0.049 U mL^− 1^ min^− 1^ at 133 h. Maximum extracellular protein concentration was observed at 122 h to reach 71.7 mg mL^− 1^ followed by reduction in its concentration. During the course of the process, colloidal chitin concentration was decreased slowly without a full consumption where its value reached 45.3 g L^− 1^ at 145 h. The total protein concentration of cells of Actino 48 was increased slowly during the lag phase and continued for 8 h. After that, the culture entered the exponential phase (log phase). The maximum total protein concentration of the cells was observed at 133 h to be 99.9 mg mL^− 1^. Chitinase activity increased over time with a production rate (*Q*_*p*_) of 0.0002 U mL^− 1^ h^− 1^ (Fig. 4B). Additionally, yield coefficient (*Y*_*p/s*_) was calculated and recorded the same value of 0.0002 (U mL^− 1^ min^− 1^) chitinase activity/g chitin (Fig. 4C). The dissolved oxygen decreased rapidly as a result of the increasing demand for O_2_ that is required for actinobacterial growth. It reached its low value of 5% at 10 h followed by increasing for 2 h. After that, the dissolved oxygen decreased to reach 6% at 19 h followed by an increase to reach 100% by the end of the fermentation process. In addition, pH was increased to reach 7 at 19 h and reached its maximum value of 8.9 at the end of the process (Fig. 5).

**Fig. 6:**
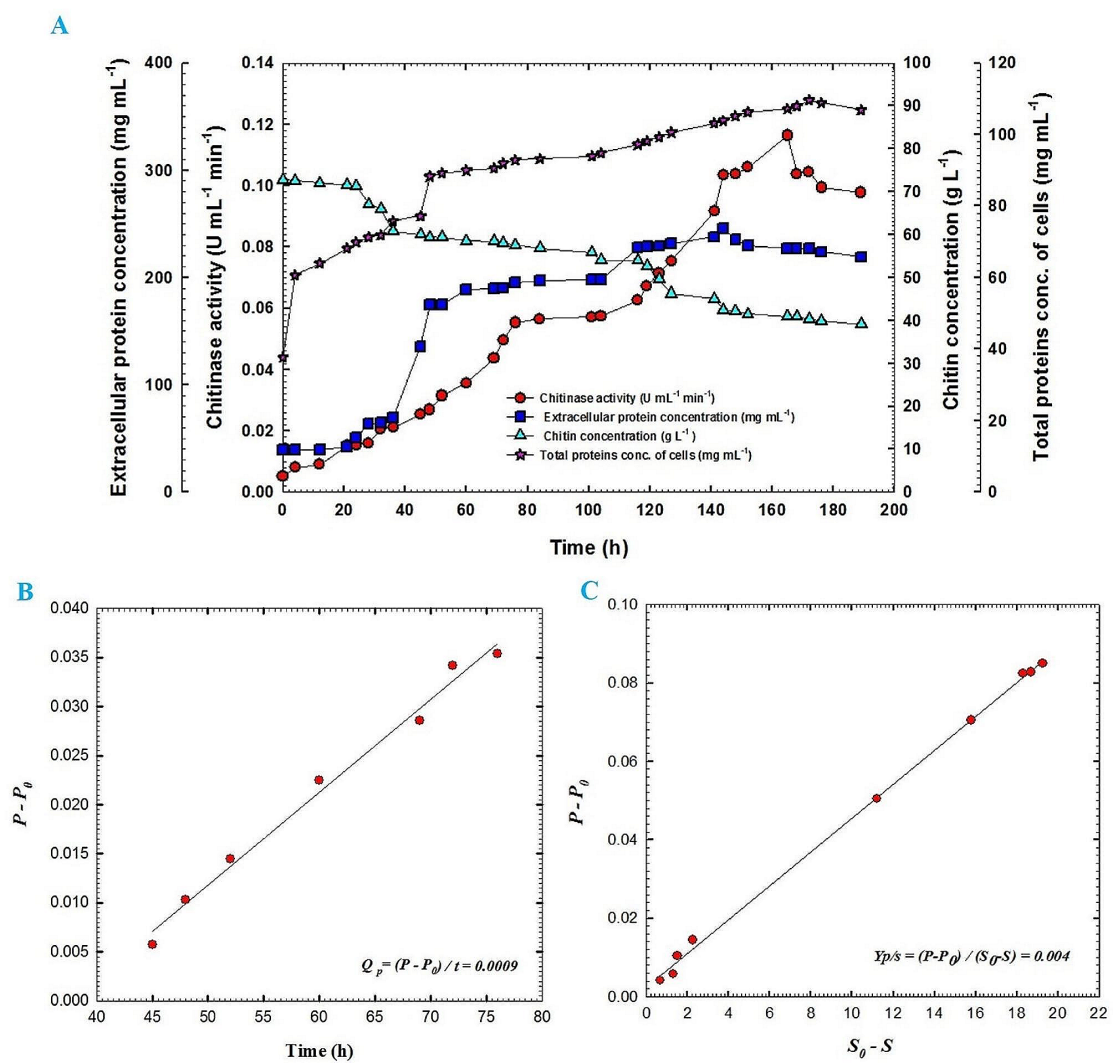
(**A**) Chitinase activity, extracellular protein concentration, chitin concentration and total protein concentration of cells of fermentation broth of *Streptomyces cellulosae* Actino 48 as a function of time for batch fermentation No. 2, (**B**) production rate of chitinase enzyme in the exponential growth phase of batch fermentation No. 2 of Actino 48 and (**C**) yield coefficient of chitinase activity of batch fermentation No. 2 of Actino 48 on chitin; P_0_ represents chitinase activity (U mL^− 1^ min^− 1^) in the broth at initial time t_0_; P, chitinase activity (U mL^− 1^ min^− 1^) in the broth at time t; S_0_, chitin concentration in the broth at initial time t_0_ (g L^− 1^) and S, chitin concentration in the broth at time t (g L^− 1^). Data were analyzed statistically at the significant level of *P* ≤ 0.05

**Fig. 7:**
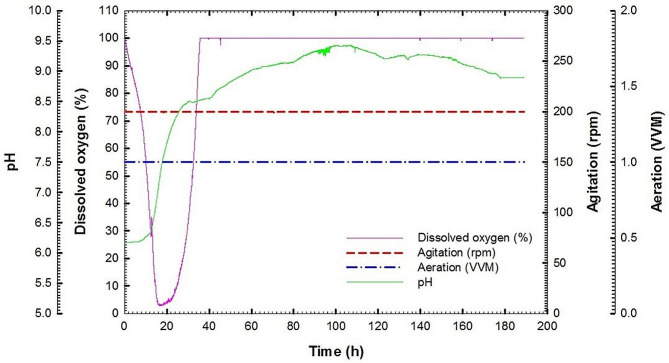
Dissolved oxygen, agitation, aeration and pH (online data) as a function of time during batch fermentation No. 2 of *Streptomyces cellulosae* Actino 48

Batch fermentation No. 2 of Actino 48 was performed at 30 °C with agitation speed and aeration of 200 rpm and 1 VVM, respectively. Chitinase activity, extracellular protein concentration, chitin concentration and total protein concentration of actinobacterial cells of culture broth for *S*. *cellulosae* Actino 48 were plotted against time. The highest chitinase activity was 0.1163 U mL^− 1^ min^− 1^ at 165 h and then the activity decreased gradually to 0.0977 U mL^− 1^ min^− 1^ at 189 h. Extracellular protein concentration increased rapidly after 36 h to record its maximum value (245.7 mg mL^− 1^) at 144 h. Colloidal chitin concentration was decreased slowly to reach 39.1 g L^− 1^ at the end of the fermentation process without complete consumption. The growth of Actino 48 was increased slowly during culture lag phase, which continued for 8 h. Maximum total protein concentration of the cell was observed at 172 h to reach 109.5 mg mL^− 1^ (Fig. 6A). Chitinase activity increased over time with *Q*_*p*_ of 0.0009 U mL^− 1^ h^− 1^ (Fig. 6B). Relationship between chitinase activity and chitin concentration as a substrate was drawn to calculate yield coefficient, which recorded 0.004 (U mL^− 1^ min^− 1^) chitinase activity/g chitin (Fig. 6C). The dissolved oxygen concentration decreased rapidly, as a result of the increasing demand for O_2_ that is required for actinobacterial growth, to reach its lowest value of 3% at 20 h followed by increasing trend to reach 100% at the end of the fermentation process. In addition, pH was increased to reach pH 7 at 16 h and reached its maximum value of pH 9.42 at 105 h. After that, the pH value decreased to reach pH 8.89 at the end of the fermentation process (Fig. 7).

**Fig. 8:**
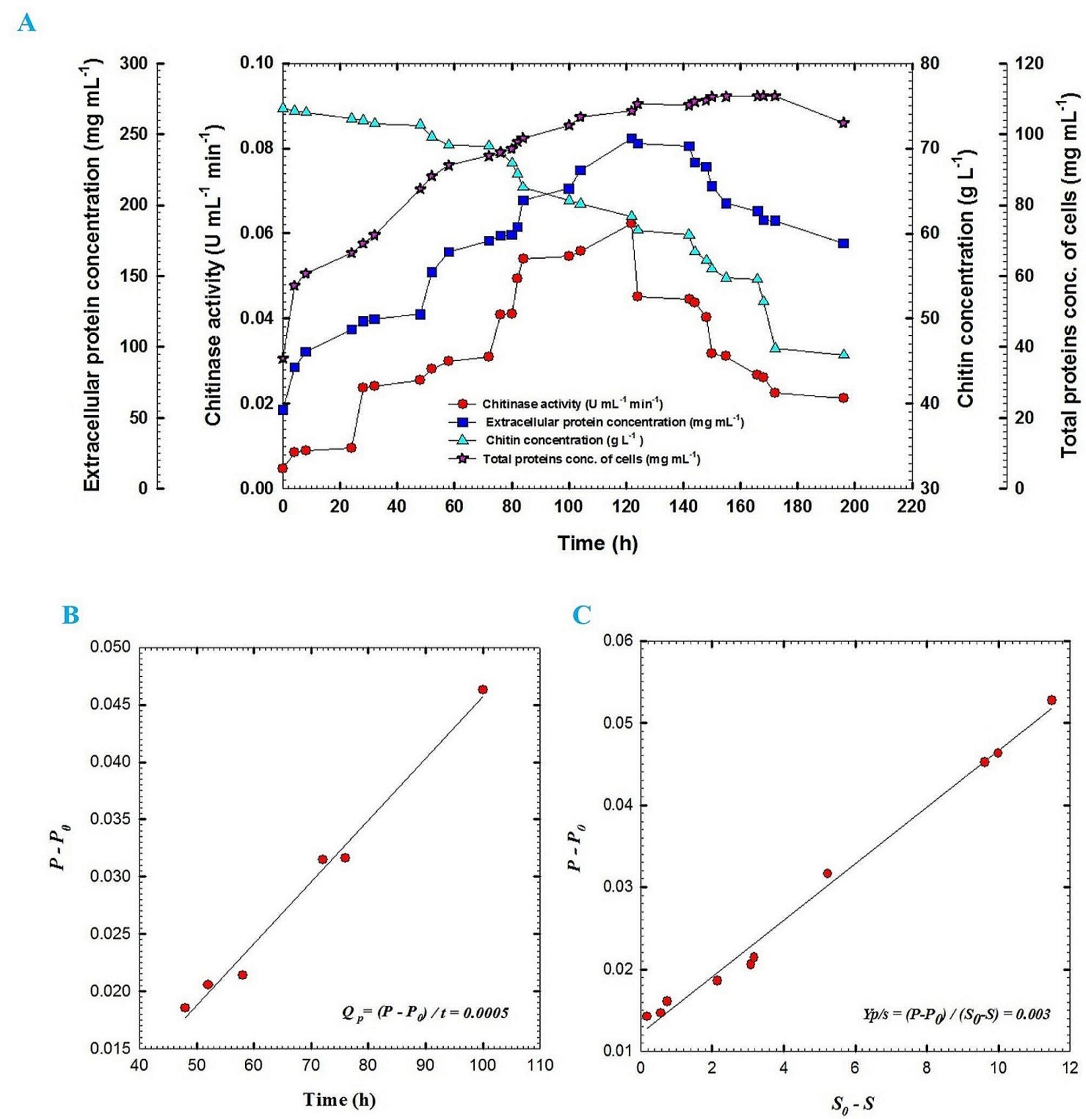
(**A**) Chitinase activity, extracellular protein concentration, chitin concentration and total protein concentration of cells of fermentation broth of *Streptomyces cellulosae* Actino 48 as a function of time for batch fermentation No. 3, (**B**) production rate of chitinase enzyme in the exponential growth phase of batch fermentation No. 3 of Actino 48 and (**C**) yield coefficient of chitinase activity of batch fermentation No. 3 of Actino 48 on chitin; P_0_ represents chitinase activity (U mL^− 1^ min^− 1^) in the broth at initial time t_0_; P, chitinase activity (U mL^− 1^ min^− 1^) in the broth at time t; S_0_, chitin concentration in the broth at initial time t_0_ (g L^− 1^) and S, chitin concentration in the broth at time t (g L^− 1^). Data were analyzed statistically at the significant level of *P* ≤ 0.05

**Fig. 9:**
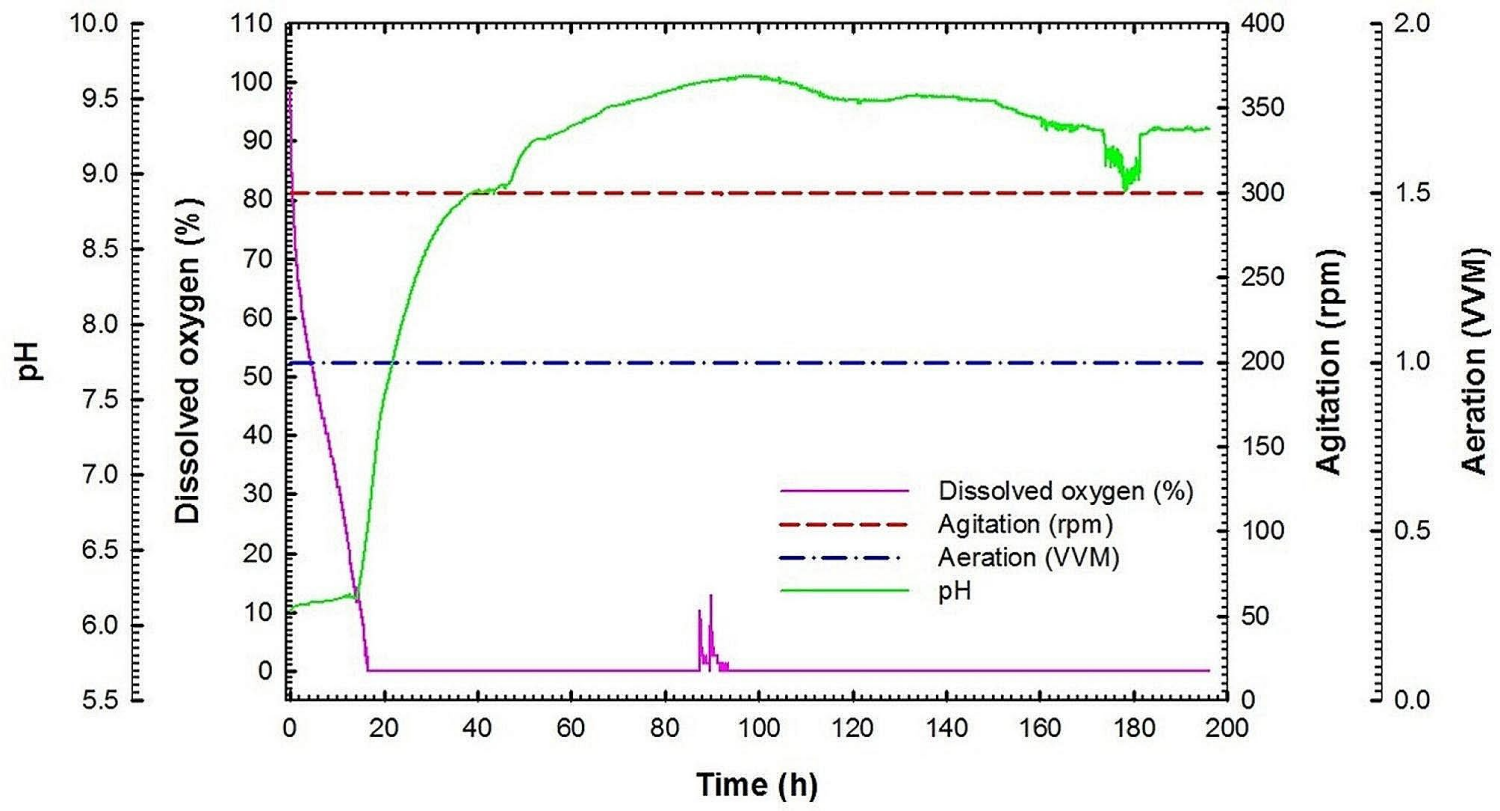
Dissolved oxygen, agitation, aeration and pH (online data) as a function of time during batch fermentation No. 3 of *Streptomyces cellulosae* Actino 48

Batch fermentation No. 3 of *S*. *cellulosae* Actino 48 was completed at 30 °C with agitation and aeration rates of 300 rpm and 1 VVM, respectively. Figure 8A shows chitinase activity, extracellular protein concentration, chitin concentration and total protein concentration of actinobacterial cells of culture broth for *S*. *cellulosae* Actino 48 as a function of time. The results indicated that the maximum activity of chitinase enzyme reached 0.0622 U mL^− 1^ min^− 1^ at 122 h then decreased to 0.0213 U mL^− 1^ min^− 1^ at 196 h. Extracellular protein concentration was increased at 24 h to reach 112.3 mg mL^− 1^ and reached its maximum value of 247 mg mL^− 1^ at 122 h. Colloidal chitin concentration was found to decrease slowly and its value reached 45.7 g L^− 1^ at the end of the fermentation process. Growth of *S*. *cellulosae* Actino 48 was increased slowly during the lag phase, which continued for 24 h. The total protein concentration of the actinobacterial cells reached its maximum value of 110.8 mg mL^− 1^ at 168 h then decreased to 103.2 mg mL^− 1^ at the end of the fermentation process at 196 h. Chitinase activity was found to increase over time with *Q*_*p*_ of 0.0005 U mL^− 1^ h^− 1^ (Fig. 8B). Yield coefficient was calculated as 0.003 (U mL^− 1^ min^− 1^) chitinase activity/g chitin (Fig. 8C). Culture was maintained at a higher value of dissolved oxygen, which decreased rapidly as a result of the increasing demand for O_2_ that is required for actinobacterial growth. Dissolved oxygen reached its low value of 0% at 16.5 h and it continued at 0% value till the end of the fermentation process. In addition, pH value was increased with observable rate until it reached 7 at 18 h and the maximum pH value was reached 9.65 at 100 h. After that, pH value was decreased to reach 9 at 180 h (Fig. 9).

#### Greenhouse experiment

Four bio-formulations were prepared using the resulted culture broth of chitinase-producing *S*. *cellulosae* Actino 48 including, talc-based formulation, bentonite-based formulation, nano talc-based formulation and nano bentonite-based formulation. Bioformulations were effective in reducing disease incidence and severity on peanut plants when they were used as biocontrol agents in a soil infested with *R. solani*. Peanut seeds were treated with each formulation and sown seven days post soil infestation. Damping-off percentage, root rot percentage, and healthy survival percentage were evaluated. Furthermore, shoot dry weight, root dry weight, and total pods dry weight were measured. Statistical analysis of the generated data was performed using ANOVA test and the results were summarized in Tables 1, 2 and 3.

**Table 1:** Effect of bioformulations of *Streptomyces cellulosae* isolate actino 48 on peanut damping- off and root rot caused by *Rhizoctonia solani* under greenhouse conditions

Treatment	Damping off (%)	Reduction (%)^Y^	Root rot (%)	Reduction (%)^Y^	Healthy survival (%)
***Rhizoctonia solani*** **(check)**	*47.62 ± 16.5 ^a^**	--------	23.81 ± 8.2 ^a^	--------	28.57 ± 14.3 ^d^
**Control**	14.29 ± 0.0 ^bc^	70.00	4.76 ± 8.2 ^bc^	80.01	80.95 ± 8.2 ^ab^
**Talc formulation**	14.29 ± 0.0 ^bc^	70.00	00 ± 0.0 ^c^	100	85.71 ± 8.2 ^ab^
**Bentonite formulation**	4.76 ± 8.2 ^c^	90.00	00 ± 0.0 ^c^	100	95.24 ± 8.2 ^a^
**Nano talc formulation**	4.76 ± 8.2 ^c^	90.00	00 ± 0.0 ^c^	100	95.24 ± 8.2 ^a^
**Nano bentonite formulation**	4.76 ± 8.2 ^c^	90.00	00 ± 0.0 ^c^	100	95.24 ± 8.2 ^a^
**TF +** ***R***. ***solani***	19.05 ± 8.2 ^bc^	60.00	14.29 ± 0.0 ^ab^	39.98	66.67 ± 8.2 ^bc^
**BF +** ***R***. ***solani***	38.10 ± 8.2 ^ab^	20.00	9.52 ± 8.2 ^bc^	61.15	52.38 ± 8.2 ^c^
**NTF +** ***R***. ***solani***	19.05 ± 8.2 ^bc^	60.00	4.76 ± 8.2 ^bc^	80.01	76.19 ± 8.2 ^ab^
**NBF +** ***R***. ***solani***	23.81 ± 8.2 ^abc^	50.00	4.76 ± 8.2 ^bc^	80.01	71.43 ± 14.3 ^bc^
**Rizolex-T +** ***R***. ***solani***	14.29 ± 0.0 ^bc^	70.00	00 ± 0.0 ^c^	100	85.71 ± 0.0 ^ab^
**LSD 0.05**	23.82	--------	13.97	--------	23.45
**F-value**	2.85	--------	2.564	--------	6.632
**P-value**	0.0194	--------	0.0315	--------	0.0001

* Means in each column followed by the same latter do not differ significantly (*P* ≤ 0.05); ** Significant letters and ^Y^ Reduction percentage (%) = [check-treatment]/check × 100

**Table 2:** Effect of different bioformulations of *Streptomyces cellulosae* isolate actino 48 on dry weight of shoot and root system of peanut plants growing in a soil infested with *Rhizoctonia solani*

Treatment	Dry weight of shoot system (g)	Increase (%)^Z^	Dry weight of root system (g)	Increase (%)^Z^
***Rhizoctonia solani*** **(check)**	*13.88 ± 1.1 ^f^**	--------	1.58 ± 1.1 ^e^	--------
**Control**	29.79 ± 0.4 ^ab^	53.41	3.20 ± 0.3 ^bcd^	50.63
**Talc formulation**	27.07 ± 1.6 ^bc^	48.73	3.11 ± 0.1 ^bcd^	49.20
**Bentonite formulation**	27.76 ± 1.1 ^bc^	50	3.72 ± 0.4 ^abc^	57.53
**Nano talc formulation**	32.53 ± 1.6 ^a^	57.33	4.47 ± 0.5 ^a^	64.65
**Nano bentonite formulation**	30.52 ± 0.9 ^ab^	54.52	4.01 ± 0.2 ^ab^	60.60
**TF +** ***R***. **solani**	17.73 ± 1.2 ^ef^	21.72	3.06 ± 0.7 ^cd^	48.37
**BF +** ***R***. **solani**	20.41 ± 1.6 ^e^	31.99	3.35 ± 0.6 ^bcd^	52.84
**NTF +** ***R***. **solani**	26.50 ± 1.3 ^bc^	47.62	3.56 ± 0.1 ^abcd^	55.62
**NBF +** ***R***. **solani**	21.27 ± 1.8 ^de^	34.74	3.35 ± 0.7 ^bcd^	52.84
**Rizolex-T +** ***R***. **solani**	25.2 ± 1.7 ^cd^	44.92	2.64 ± 0.4 ^d^	40.15
**LSD 0.05**	4.50	--------	0.92	--------
**F-value**	14.260	--------	5.681	--------
**P-value**	0.0000	--------	0.0003	--------

* Means in each column followed by the same latter do not differ significantly (*P* ≤ 0.05); ** Significant letters and ^Z^ Increase percentage = [treatment-check]/treatment × 100

**Table 3:** Effect of different bioformulations of *Streptomyces cellulosae* isolate actino 48 on the number of total peanut pods and yield increase in a soil infested with *Rhizoctonia solani* under greenhouse conditions

Treatment	No. of total pods	Yield increase (%)^Z^
***Rhizoctonia solani*** **(check)**	*8.67 ± 0.6 ^i^**	--------
**Control**	15.00 ± 1.0 ^de^	42.2
**Talc formulation**	16.67 ± 0.6 ^c^	47.99
**Bentonite formulation**	17.68 ± 1.2 ^bc^	50.96
**Nano talc formulation**	19.67 ± 0.6 ^a^	55.92
**Nano bentonite formulation**	18.67 ± 1.2 ^ab^	53.56
**TF +** ***R***. ***solani***	12.33 ± 0.6 ^gh^	29.68
**BF +** ***R***. ***solani***	11.65 ± 0.6 ^h^	25.58
**NTF +** ***R***. ***solani***	14.00 ± 1.0 ^ef^	38.07
**NBF +** ***R***. ***solani***	13.33 ± 1.2 ^fg^	34.96
**Rizolex-T +** ***R***. ***solani***	16.33 ± 0.6 ^cd^	46.91
**LSD 0.05**	1.44	--------
**F-value**	44.925	--------
**P-value**	0.0000	--------

* Means in each column followed by the same latter donot differ significantly (*P* ≤ 0.05);** Significant letters and ^Z^ Increase percentage = [treatment-check]/treatment × 100

Four bio-formulation used in this study were effective in reducing the damping-off caused by *R. solani* compared to the control treatment. Treatment with *R*. *solani* showed a significant damping-off percentage (47.62%) at the significant level of *p* ≤ 0.05 (*p*-value, 0.0149). Treatments using talc and nano talc formulations of *S*. *cellulosae* Actino 48 in soil infected with *R*. *solani* showed a significant effect in reducing damping-off percentage, which was decreased to reach 19.05%, with a reduction percentage of 60% for both formulations. Moreover, Rizolex-T, a fungicide, reduced the damping-off percentage to 14.29% with a reduction percentage of 70% with no significant differences between each other. The percentage of root rot caused by *R*. *solani* reached 23.81% (*p*-value, 0.0315), while treatments using nano talc and nano bentonite formulations of Actino 48 isolate in soil infected with *R*. *solani* were effective in reducing root rot percentage (4.76%) with a reduction percentage of 80.01% for both formulations. The percentage of healthy survival peanut plants in the case of the treatments using nano talc formulation of Actino 48 and Rizolex-T (standard) in soil infected with *R*. *solani* increased to reach 76.19% and 85.71%, respectively. Moreover, this percentage reached 28.57% in the treatment by *R. solani* alone at the significant level of *p* ≤ 0.05 (*p*-value, 0.0001) (Table 1).

The results indicated that all the formulations stimulated the growth of peanut plants in soil infected with *R. solani* compared to the other control treatment. Dry weights of shoots and roots were increased significantly in the treatments with bio-formulations of Actino 48 in soil infected with *R. solani*. The nano talc formulation and Rizolex-T were more significant in increasing the dry weight of the peanut plant shoots in soil infected with *R*. *solani* than other treatments. The dry weight of the peanut plant shoots reached 26.5 g and 25.2 g with an increasing percentage of 47.62% and 44.92%, respectively. On the other hand, treatment with *R*. *solani* alone decreased the dry weight of the shoots to reach 13.88 g. Additionally, treatment with nano talc formulation of Actino 48 in the *R*. *solani* infected soil increased the root dry weight of the treated peanut plants significantly to reach 3.56 g with an increasing percentage of 55.62% followed by the nano bentonite formulation treatment, which achieved 3.35% with an increased percentage of 52.84%. Meanwhile, treatment with *R*. *solani* alone decreased the root dry weight of the treated plants to reach 1.58 g (Table 2).

The number of total pods recorded in the *R*. *solani* treatment was 8.67. On the other hand, treatment with Rizolex-T in soil infected with *R*. *solani* achieved a maximum number of total pods of 16.33 with a yield increase percentage of 46.91%. Also, talc, bentonite, nano talc, and nano bentonite formulations of *S*. *cellulosae* in soil infected with *R*. *solani* increased the number of total pods to record 12.33, 11.65, 14, and 13.33, respectively with maximum yield increase percentages. Further, nano talc formulation of *S*. *cellulosae* Actino 48 achieved the highest percentage of yield increase that reached 38.07% (Table 3).

## Discussion

Peanut is one of the major edible oil production crops around the world. Additionally, peanut is an important food and feed crop because it is rich in fats, proteins, minerals, phenolic compounds and vitamins [13]. Spores-producing bacteria is considered an alternative approach to chemical pesticides for controlling plant diseases that can be formulated and utilized as biocontrol agents [14]. Actinobacteria play an important role as biocontrol agents of plant diseases. Currently, various formulations of chitinase-producing *Streptomyces* spp. are used as active bio-fungicides to complete the biocontrol processes [15]. The mode of action of antagonistic *Streptomyces* producing chitinase enzymes is based on the cell wall degradation of phytopathogenic fungi that mainly composed of chitin.

In our investigation, we evaluated thirteen isolates of actinobacteria as potential biocontrol agents against the mycelial growth of *R*. *solani in vitro*. Among these isolates, Actino 48 exhibited superior inhibitory activity against *R*. *solani* mycelia compared to the other actinobacterial isolates. These results are reliable with the research conducted by Sadeghi et al. (2006) [9], which showed that two chitinolytic streptomycetes had greater efficacy in inhibiting the mycelial growth of *R*. *solani*, the pathogen responsible for sugar beet damping-off disease. Furthermore, Abdel-Gayed et al. (2019) [16] stated that *B*. *subtilis* strain B4 exhibited the strongest inhibitory impact on *R*. *solani* in peanut, leading to a greater diameter of the inhibition zone compared to the other strains.

Production of chitinase-producing *Streptomyces* spp. using fermentation technology is the primary step toward their formulations and commercial applications. Agitation is a vital factor affecting the production of chitinase enzyme from *Streptomyces* spp. Liu et al. (2003) [17] reported that the production of chitinase is facilitated with a certain agitation rate. Mixing and shearing in the fermentation process mainly occur by stirring, which can make oxygen, heat and nutrients mix fully, and be transferred efficiently in the fermentation broth. Agitation disperses the air into small bubbles to improve the gas-liquid contact area, and prevent mycelia from clustering in favor of oxygen absorption [18]. Increasing the agitation speed may result in the formation of uneven mixing and shear forces that have the potential to harm delicate microorganisms and impact the formation of the final product [19]. Conversely, the lowest agitation speed will increase the viscosity of the fermentation broth, which leads to a decrease in mass transfer efficiency [20]. In the current investigation, three agitation rates were utilized to complete the fermentation process. Our results showed batch fermentation process No. 2 in the bioreactor, which was performed at an agitation speed of 200 rpm, gave a higher production of chitinase enzyme compared to batch fermentation processes No. 1 and No. 3, which were carried out at an agitation speed of 150 and 300 rpm, respectively. Batch fermentation process No. 2 produced a maximum production of chitinase enzyme that reached 0.1163 U mL^− 1^ min^− 1^ with a production rate *Q*_*p*_ of 0.0009 U mL^− 1^ h^− 1^ but batch fermentation process No. 1 and No. 3 produced 0.049 U mL^− 1^ min^− 1^ (*Q*_*p*_, 0.0002 U mL^− 1^ h^− 1^) and 0.062 U mL^− 1^ min^− 1^ (*Q*_*p*_, 0.0005 U mL^− 1^ h^− 1^), respectively. Zhou et al. (2018) [21] produced a maximum production of glycoprotein GP-1 from *S*. *kanasenisi* ZX01 at an agitation speed of 200 rpm compared to 150, 250 and 300 rpm, which produced a low concentration of glycoprotein GP-1. They revealed that the higher agitation speeds of 250 and 300 rpm could cut off mycelium and damage the cell structure owing to unbearable shear force. Zambry et al. (2021) [22] documented that the agitation speed of 200 rpm via batch fermentation process in the stirred tank bioreactor achieved maximum production of biosurfactant from *Streptomyces* sp. compared to agitation speeds of 400 and 600 rpm. However, the reality of the effects of agitation rate needs to be further elucidated since the biosynthesis of chitinase seems to be compromised with a pH or cultivation environment [23].

*R*. *solani* have several weapons that make its control very difficult. *R*. *solani* is a soil-borne pathogen and have a wide host range. It is made of sclerotia, so it can survive in the soil for unlimited times. Chemical control of *R*. *solani* was not very effective and most of the chemicals caused hazardous effects on non-targeted organism and the environment [24]. Due to the increased consumer concern regarding chemical pesticides residues in foods and environmental safety, there is an increasing demand for developing alternative methods, such as biocontrol [25]. Actinobacteria are considered potential biocontrol agents of plant diseases. The soil is a good source of *Streptomyces* species, which have antagonistic activity against different soil-borne pathogens such as *R*. *solani* [26–28] The efficacy of actinobacterial isolates in the biocontrol of the *R*. *solani* damping-off disease was studied and highlighted by Patil et al. (2011) [24]. Martinez-Alvarez et al. (2016) [14] reported that spores-producing bacteria can be used as an alternative means to chemical pesticides in controlling plant diseases. Several modes of actions of actinobacteria involved in the biocontrol of plant pathogens were suggested such as production of antibiotic compounds, siderophores, hydrogen cyanide (HCN) and hydrolytic enzymes such as chitinases and glucanases [29–31]. Additionally, *Streptomyces* species play an important role in biocontrol of soil borne pathogens via induced systemic resistance (ISR) [32, 33]. In the present study, four wettable powder formulations (talc, bentonite, nano talc and nano bentonite) of chitinase-producing *S*. *cellulosae* isolate Actino 48, which were used as biocontrol agents in a soil infected with *R*. *solani*, were efficient in decreasing damping-off and root rot of peanut. A wettable talc powder formulation of *S*. *rochei* strain PTL2 has been recorded as biofungicide, which generated chitinase and other substances, was successful in the biological control of *R*. *solani* [34]. Zacky and Ting (2015) [15] formulated chitinase-producing *Streptomyces* spp. and employed this formulation as a biofungicide in the biocontrol of certain plant pathogenic fungi. Chitinolytic *S*. *vinaceusdrappus* S5MW2 played an important role in the biocontrol of *R*. *solani* under greenhouse conditions [11]. Formulations of *S*. *tritolerans* ARK 17 and *S*. *manipurensis* ARK 94 were more effective in reducing root rot disease of soybean compared to another formulations [28]. Our findings in this study showed that chitinase-producing *S*. *cellulosae* Actino 48 formulations effectively enhanced the dry weights of peanut plant shoots and roots. The yield of the infected plants treated with formulations of chitinase-producing Actino 48 significantly increased compared with the untreated infected plants and that treated with the chemical fungicide. Our results agree with the outcomes data reported by Abo-Zaid et al. (2021) [35]. These results may be due to the ability of *S*. *cellulosae* Actino 48 to produce some phytohormones such as auxin, cytokinin, and gibberellins. The results reported by Khamna et al. (2010) [36] and Goudjal et al. (2013) [37] showed that actinobacteria improved the germination of seeds and elongation of roots as a result of the ability of phytohormones production. Zamoum et al. (2017) [34] stated that the PTL2 strain of *S*. *rochei* increased the dry weight of the tomato plant, as it had the capacity to generate plant hormones such as IAA and GA3. Xiao et al. (2002) [38] documented that treatment the plant with *Streptomyces* sp. CA-2 and AA-2 boosted the plant growth, which made the plant more protected against phytopathogenic fungi. Finally, our results of this investigation show that *S*. *cellulosae* Actino 48 is a promising microorganism and in additions to being an excellent to bio-control agent it can also be utilized as a plant growth promoting rhizobacteria.

## Conclusions

Actino 48 showed the highest inhibition percentage of mycelium growth of *R*. *solani*, which reached 97%. Batch fermentation No. 2 of Actino 48 achieved high chitinase activity of 0.1163 U mL^− 1^ min^− 1^ with a yield coefficient of 0.004 U mL^− 1^ min^− 1^ chitinase activity/g chitin. Nano-talc formulation of Actino 48 had more a significant effect compared to the other formulations in reducing percentages of damping-off and root rot diseases caused by *R*. *solani* that equal to 19.05% and 4.76% with reduction percentages of 60% and 80%, respectively. Furthermore, the nano-talc formulation of Actino 48 was sufficient in increasing the dry weight of the peanut plants shoot, root systems, and the total number of peanut pods with increasing percentages of 47.62%, 55.62%, and 38.07%, respectively. The ongoing inquiry demonstrated that the Actino 48 isolate of *S*. *cellulosae* is regarded as a hopeful actinobacterial isolate in managing fungal soil-borne diseases and can be exploited as a biocontrol agent.

## Materials and methods

### Fungal and actinobacterial isolates

The fungus isolate utilized in the current study was obtained from Behira governorate in Egypt and isolated from peanut. The fungus isolate was identified as *R*. *solani* based on morphological characteristics by the staff of fungi lab. at the Agricultural Research Center (Giza, Egypt). The pathogenic potential of this isolate was confirmed. Thirteen actinobacterial isolates were obtained from City of Scientific Research and Technological Applications (SRTA-City) (Alexandria, Egypt). All actinobacterial isolates were obtained from Behira governorate in Egypt. Actinobacterial isolates were isolated from rhizosphere of cauliflower (Actino 23 and 24), eggplant (Actino 27), corn (Actino 29), pepper (Actino 32, 34 and 38), cotton (Actino 42 and 43), red pepper (Actino 46 and 47) and molokhia (Actino 48 and 49). Through analysis of the 16 S rDNA sequence, the noteworthy actinobacterial strain Actino 48 was identified as *S*. *cellulosae* and was submitted to GenBank with the accession number MT573878.

### Antagonistic effect of actinobacterial isolates against *R. solani*

Biomass development of *R*. *solani* was estimated in the existence of actinobacterial isolates to determine their antagonistic effect according to Trivedi et al. (2008) [39] with slender amendments. One mL of the 5-day-old pre-culture of actinobacterial isolates was introduced into 50 mL potato dextrose broth (PDB) containing *R*. *solani* plug (6 mm diameter) from newly cultivated culture on potato dextrose agar (PDA). Incubation of cultures was performed for seven days at 30 ºC. A control flask was utilized, which contained PDB with solely the fungal plug. The materials from the flasks were sieved through pre-measured Whatman No. 1 filter paper and permitted to desiccate at 50 ºC. Inhibition percentage of the fungal growth was defined as the percentage of weight loss of the examined fungus in the presence of the antagonistic bacteria that was assessed using the equation: (W1 - W2) / W1 × 100, where W1 signifies the weight (g) of the tested fungus in a control flask and W2 is the weight of the fungus in the existence of combatant bacteria (g).

### Assessment of chitinase production by actinobacterial strains through qualitative and quantitative means

#### Detection of chitinase production

Actinobacterial cultures were inoculated onto agar plates containing colloidal chitin and kept at 30 ºC for 10 days. The improvement of a clear area surrounding the colony validated the synthesis of chitinase.

#### Chitinase assay

To perform the chitinase test, we cultivated fresh cultures of each actinobacterial isolate (10^7^ CFU mL^− 1^) in a minimal liquid medium (MLM) supplemented with colloidal chitin (1% *w: v*) (LOBA Chemie PVT. LTD., Maharashtra, India). The MLM contained (g L^− 1^) MgSO_4_.7H_2_O, 0.2; K_2_HPO_4_, 0.9; KCl, 0.2; NH_4_NO_3_, 1.0; FeSO_4_.7H_2_O, 0.002; MnSO_4_, 0.002; ZnSO_4_, 0.002; pH 6.8. The cultures were incubated for 8 days at 30 ºC in flasks. We used the Boller and Mauch 1988 [40] method to estimate chitinase levels every day. To perform the colorimetric test, we mixed 1 mL of cell-free supernatant with 1 mL of colloidal chitin (1% *v: v*) in a citrate phosphate buffer (0.1 M pH 6.5) and incubated the mixture at 40 ºC for 2 h in a shaking water bath. We stopped the reaction by adding 2 mL of DNS reagent and kept it in a boiling water bath for 5 min to develop the color. After cooling, we centrifuged the tubes at 5000× g for 10 min and measured the absorbance at optical density (OD) 575 nm against the blank. We determined one unit of chitinase as the quantity of enzyme that liberates 1 µmoL of N-acetylglucosamine per minute under the reaction circumstances.

### Detection of interaction between actinobacterial isolate Actino 48 and *R. solani*

A scanning electron microscope (SEM, JEOL JSM-6360LA, Tokyo, Japan) was employed to observe and examine the inhibitory interaction between *R*. *solani* and the potential actinobacterial isolate Actino 48, which was recognized as *S*. *cellulosae*. A dual-culture agar plate assay was utilized to detect the prior interaction.

### Fermentation experiments

#### Bioreactor

A 10-L bench-top bioreactor (Cleaver, Saratoga, USA) with three six-bladed disk-turbine impellers and four baffles was employed for batch fermentation. The bioreactor was connected to a digital control unit and the process was automated using a control panel with a 10.4 color touch screen interface and 59.994 programs for various conditions. The temperature was set at 30 °C and pH was not controlled. Compressed air was filtered and regulated to 1 VVM (air volume per broth volume per minute). The online monitoring of dissolved oxygen and pH values was carried out using METTLER TOLEDO electrodes. To eliminate the foam generated, antifoam (Sigma) was added.

#### Batch fermentation

Three batch fermentation processes were carried out in the bioreactor with various agitation speed, which was 150, 200 and 300 RPM. Any batch fermentation process of *S*. *cellulosae* Actino 48 was initiated in the bioreactor by inoculation in production medium containing in g L^− 1^ colloidal chitin, 75; peptone, 20; yeast extract, 0.5; KNO_3_, 0.2; MgSO_4_.7H_2_O, 0.03; KCl, 2.5; K_2_HPO_4_, 1; FeSO_4_.7H_2_O, 0.01; ZnSO_4_.7H_2_O, 0.001; MnSO_4_.7H_2_O, 0.001. For every batch fermentation process, several samples of culture broth were taken to measure the activity of chitinase enzyme, protein content of the supernatant, chitin consumption and biomass determination.

### Analytical procedures

#### Chitinase assay

The sample was centrifuged and the activity in the supernatant (crude enzyme) was assayed as previously mentioned [40].

#### Quantitative assay of protein

Assay for total protein concentration was determined according to Bradford (1976) [41] with bovine serum albumin (Sigma) as a standard.

### Determination of colloidal chitin consumption

The colloidal chitin was determined in every sample to follow up its consumption as the main carbon source using the following method; 1 mL of sample was added to 3 mL of concentrated H_2_SO_4_ and autoclaved for two hours at 121˚ C to convert the chitin to its reducing monomer then centrifuged to get a clear solution. Later, 3 mL of DNS was added to 2 mL of clear solution and boiled for 10–15 min. After cooling, the absorbance was measured at 540 nm [42]. A standard curve was plotted using different concentrations of colloidal chitin as a standard.

### Formulation of culture broth of chitinase-producing *S. cellulosae* Actino 48

Culture broth of the antagonistic chitinase-producing Actino 48, which exhibited a great inhibition proportion against *R*. *solani* compared to other actinobacterial isolates, was used for the preparation of a bioformulation to reduce peanut soil-borne diseases. Talc powder (T) and bentonite (B) in a natural and nano form were used as carriers for the preparation of bio-friendly formulations. We added 10 g of colloidal chitin as a carbon source and an adhesive agent to 400 mL of culture broth (including 10^7^ CFU mL^*−* 1^ of *S*. *cellulosae* Actino 48). The mixture with supplements was uniformly blended in a vortex mixer. To attain a pH of 7.0, we incorporated 15 g of calcium carbonate into 1 kg of sterilized talc powder and bentonite that combined seamlessly. A blend of 400 mL of supplemented culture broth with 1 kg of talc powder and bentonite was prepared. The moisture level of the bioformulation was reduced to below 20% by desiccation, and it was refrigerated at 4 ºC until needed [43].

### Implementation of environmentally-friendly bio-formulations of chitinase-producing Actino 48 as biocontrol agents against *R. solani* on peanut

### Preparation of fungal inoculum

A growth medium consisting of sorghum, rough sand, and water (in a volume ratio of 2:1:2) was created to inoculate *R*. *solani*. The medium was sterilized before being inoculated with agar discs taken from the edge of a 4-day-old colony of the fungus being tested. The inoculated media were then placed in an incubator at 28 ºC for a period of 2 weeks and later utilized for soil infestation [44].

### Soil infestation

The type of soil used in the current study was sandy loam (77% sand, 11% silt and 12% clay; pH 7.98). Soil was sterilized by autoclaving for 1 h at 1.5-air pressure (121 °C). A 2% (w/w) of *R*. *solani* inoculum was introduced to the soil surface of every pot and then covered with a fine layer of sterilized soil. The infested pots were watered and left for a duration of 14 days before planting.

### Implementation dose of environmentally-friendly bio-formulations and suggested fungicide

The peanut seeds (Giza 6 cv.) were subjected to seed dressing using Actino 48, which produces chitinase, in talc and bentonite formulations, with a dosage of 10 g per kilogram of seeds. Alternatively, Rizolex-T 50% WP was used at a rate of 3 g per kilogram of seeds. The formulations were reapplied twice, as a soil drench, at a rate of 3 kg per acre, 30 and 50 days after the seeds were sown.

### Greenhouse experiment

Greenhouse experiment was carried out in New Borg El Arab city, which is located in Alexandria Governorate, Egypt, at latitude 30° 50’ 56” North, longitude 29° 36’ 42” East. Pots (25 × 25 cm diameter) containing sterilized soil were infested as mentioned above. Eleven treatments were performed as the following: (1) *R*. solani; (2) untreated healthy control; (3) talc formulation (TF); (4) bentonite formulation (BF); (5) nano-talc formulation (NTF); (6) nano-bentonite formulation (NBF); (7) TF + *R*. solani; (8) BF + *R*. *solani* (9) NTF + *R*. *solan*i; (10) NBF + *R*. solani and (11) Rizolex-T 50% WP. Seven peanut seeds (Giza 6 cv.) were treated and sown per pot. Three replicates (pots) were used for each treatment.

### Disease assessments

Disease was evaluated according to Hussien et al. (2012) [44]. The percentage of damping-off (pre- and post-emergence) was estimated 15 and 45 days after sowing using the following formulae:

% Pre-emergence = No. of non-emerged seedlings/No. of sown seeds *×* 100,

% Post-emergence = No. of dead emerged seedlings/No. of sown seeds *×* 100,

% Damping-off = pre-emergence % + post-emergence %.

Percentages of plants infected by root rot and surviving healthy plants were estimated after uprooting (120 days from sowing) as follows:

% Root-rot = No. of plants showing root rot/No. of sown seeds *×* 100,

% Apparently healthy plants = No. of surviving healthy plants/No. of sown seeds *×* 100.

Individually potted flora were uprooted and flipped over according to the ideal level of ripeness. Seed pods were separated by threshing, allowed to dry naturally for a fortnight and recorded for weight.

### Statistical analysis

Complete randomized design (CRD) was used for the analysis of the obtained results (a one-way ANOVA) using CO-STAT software (version 6.311, 2005, USA). The Least Significant Difference (L.S.D.) test was used at the 0.05% level of significance using CO-STAT program.

## Data Availability

The original contributions presented in the study are included in this article. Further inquiries can be directed to the corresponding author.
